# Alpha-1 Antitrypsin Deficiency-Associated Chronic Obstructive Pulmonary Disease

**DOI:** 10.3390/medicina62040639

**Published:** 2026-03-27

**Authors:** Evangelia Fouka, Argyro Vrouvaki, Marina Moustaka Christodoulou, Stelios Loukides, Georgios Hillas

**Affiliations:** 2nd Respiratory Medicine Department, Medical School, National and Kapodistrian University of Athens, Attikon University Hospital, 124 62 Rimini, Greece

**Keywords:** Alpha-1 antitrypsin deficiency, chronic obstructive pulmonary disease, *SERPINA1* gene, emphysema, protease–antiprotease imbalance, augmentation therapy

## Abstract

Alpha-1 antitrypsin deficiency (AATD) is a genetic disorder characterized by reduced circulating levels and/or impaired function of alpha-1 antitrypsin (AAT), a key serine protease inhibitor, in which loss of effective antiprotease protection results in unchecked neutrophil elastase activity and progressive lung tissue destruction. Although AATD accounts for approximately 1% of chronic obstructive pulmonary disease (COPD) cases and up to 2% of emphysema, AATD-related COPD remains largely underdiagnosed, despite guideline recommendations for systematic evaluation in patients with COPD, particularly in high-risk clinical settings. Pathologically, AATD-related COPD is not limited to the typical early-onset, lower-lobe-predominant emphysema, also including upper-lobe or mixed emphysema patterns, airway-predominant disease, small airways dysfunction, and bronchiectasis. Clinically, AATD-related COPD is distinguished from smoking-related COPD by its earlier onset, physiological impairment that is often disproportionate to smoking exposure, and its potential presence of certain extrapulmonary manifestations. Diagnosis and monitoring are also challenged by the frequent discordance between airflow limitation and gas transfer impairment, as well as the early involvement of small airways, limiting reliance on spirometry alone. A multimodal assessment incorporating more sensitive functional techniques and CT densitometry may provide a more precise evaluation of disease burden, progression, and prognosis. Management generally follows standard COPD principles, with intravenous AAT augmentation therapy remaining currently the only established disease-modifying therapy for selected patients with severe deficiency. The advent of new pharmacological and gene-based therapies emphasizes the importance of developing personalized management strategies that integrate genotype and longitudinal disease behavior. This narrative review summarizes current evidence on AATD-associated COPD, focusing on its genetic basis and pathophysiological features, clinical and functional heterogeneity, current and emerging diagnostic and monitoring approaches, and disease-specific management considerations.

## 1. Introduction

The current Alpha-1 antitrypsin (AAT), primarily synthesized in the liver, and, to a lesser extent, produced locally by macrophages, bronchial epithelial cells, and monocytes, is the prototypical circulating serine protease inhibitor (serpin) that predominately reaches peripheral tissues through diffusion from the circulation [1]. Its main function is tissue protection from proteolytic damage caused by serine proteinases, particularly neutrophil elastase (NE), as well as proteinase 3, and cathepsin G [2]. Under physiological conditions, tissue integrity depends on a finely regulated balance between proteases and antiproteases, largely determined by adequate circulating AAT levels, with experimental data historically suggesting “a protective threshold” of approximately 11 μM (≈0.57 g/L), based on observations correlating circulating AAT levels with reduced risk of emphysema development [3]. However, this threshold represents a population-based estimate rather than an absolute biological cutoff and does not fully account for genotype-specific variability in disease susceptibility.

A1-antitrypsin deficiency (AATD) was accidentally identified as an electrophoretic anomaly more than six decades ago and was subsequently recognized as a distinct cause of early-onset emphysema in individuals with minimal or no smoking exposure [4,5]. The condition affects approximately one in 2500–4500 individuals, being most prevalent among populations of Northern and Western European ancestry [6]. Despite its global distribution, AATD remains markedly underdiagnosed, with fewer than 10% of affected individuals identified [7]. Beyond emphysema, the disorder encompasses a range of pulmonary manifestations, including chronic bronchitis, bronchiectasis, and asthma-like features, whereas may also exhibit extrapulmonary involvement, such as liver cirrhosis, hepatocellular carcinoma, panniculitis, and systemic vasculitis [8,9].

Although significant progress has been made in the understanding of AATD-related COPD, significant uncertainty still exists regarding phenotypic heterogeneity, disease burden in heterozygous genotypes and the optimal criteria for risk stratification and timing of intervention [10,11]. Additionally, recent advances in the therapeutic landscape underscore the need for a paradigm shift towards more individualized treatment management, integrating the biological and clinical complexity of the disease [12].

In this narrative review, we provide a comprehensive overview on AATD-related COPD, highlighting current evidence on the genetic basis of the disease, its clinical expression across different phenotypes, and the factors that determine functional decline over time. A structured literature search was conducted in PubMed/MEDLINE, Embase, and Cochrane Library databases up to January 2026. Relevant studies were identified using combinations of keywords including “alpha-1 antitrypsin deficiency”, “COPD”, “emphysema”, “SERPINA1”, and “augmentation therapy”. Priority was given to international guidelines, large registry studies, randomized controlled trials, and high-quality observational studies, with emphasis on their methodological robustness and clinical relevance. Recent advances in diagnostic and treatment options are also discussed in the clinical context of a more individualized approach to the management of this genetic form of COPD.

## 2. Genetic Basis and Pathophysiology in AATD

Alpha-1 antitrypsin deficiency (AATD) is an autosomal codominant disorder of the *SERPINA1* gene, located on chromosome 14q32.1. More than 200 allelic variants have been described, which have been associated with differences in both circulating levels and AAT protein functionality [13]. The normal M allele predominates in the general population (85–90%), while pathogenic variants, such as the common Z (Glu342Lys) and S (Glu264Val) alleles are associated with reduced AAT serum concentrations [14].

When AAT is defective, absent, or at reduced levels, the consequent underregulated activity of NE within the lungs leads to increased degradation of elastin and other extracellular matrix components, resulting in alveolar destruction and emphysema development [15]. Moreover, beyond its anti-protease role, AAT exhibits significant anti-inflammatory and immunomodulatory properties, including modulation of neutrophil chemotaxis, cytokine release, and oxidative stress. Loss of these functions further amplifies airway inflammation and perpetuates proteolysis and structural damage [16,17].

**Figure 1:**
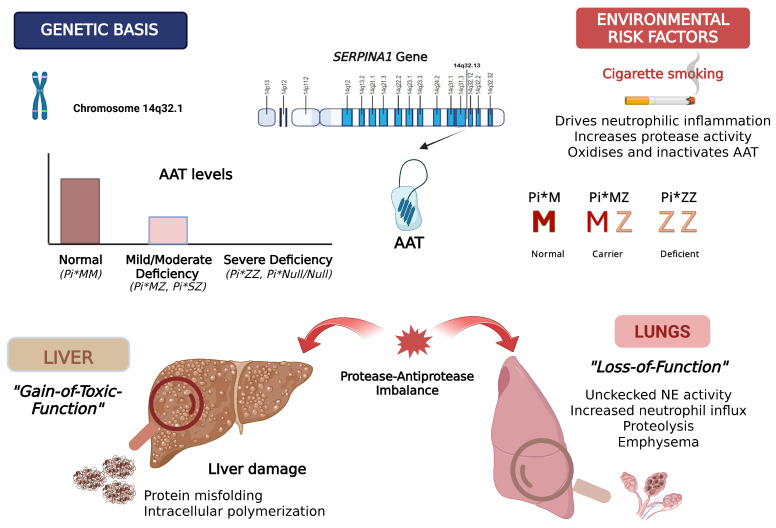
Pathophysiology of Alpha-1 Antirtypsin deficiency: Pathogenic *SERPINA1* variants result in reduced circulating AAT levels and impaired antiprotease activity. In the lungs, AATD leads to unchecked neutrophil elastase activity, proteolysis, and emphysema (“loss-of-function”). In parallel, misfolded Z-AAT accumulates within hepatocytes, causing intracellular polymerization and liver injury (“gain-of-toxic-function”). Cigarette smoking exacerbates disease expression by enhancing neutrophilic inflammation and oxidative inactivation of AAT, amplifying protease–antiprotease imbalance. Created in BioRender. Fouka, E. (31 January 2026) https://BioRender.com/ivt5csz. AAT: Alpha-1 Antirtypsin.

Patients’ genotype determines serum AAT levels and likely affects associated lung pathologies. In individuals with Null/Null genotypes, AAT is absent; however, these mutations are rare, and their clinical manifestations remain poorly characterized [14,18]. Among the deficient variants, the Z allele is responsible for abnormal protein folding, leading to intracellular polymerization and retention within hepatocytes [19,20]. Therefore, homozygosity for the Z allele (Pi*ZZ) results in severe AAT deficiency, with circulating levels typically below the so-called “protective threshold” (5–6 μmol/L) [19,21]. However, Z-polymers are also found extracellularly in the lung interstitium, where they can act as chemoattractants for neutrophils, amplifying interleukin-8 (IL-8) production and promoting elastin degradation [22,23]. This represents a potential dual pathogenic mechanism: a “gain-of-toxic-function” in the liver, due to intracellular polymer accumulation and endoplasmic reticulum stress, and a “loss-of-function” in peripheral tissues, due to a significant loss of antiprotease protection [24] (Figure 1).

Environmental exposures, particularly cigarette smoking, significantly modify disease expression across AATD genotypes [25]. Smoking represents a critical modifiable risk factor as it induces neutrophil-driven inflammation and directly impairs AAT function via oxidation and inactivation, exacerbating protease–antiprotease imbalance [25,26]. Even in milder Pi*SZ and Pi*MZ variants, smoking consistently elevates disease risk compared with ever-smoking Pi*MM or Pi*MS controls; however, the specific impact of former smoking remains less clearly defined, with lung function decline depending on established airflow obstruction and cumulative smoking exposure at cessation [27,28,29]. Notably, never-smoking Pi*MZ individuals do not appear to carry excess risk for developing airway obstruction, suggesting that genotype–environment interaction is more clinically relevant rather than AAT levels alone in determining pulmonary outcomes [29]. However, Pi*MZ individuals may still be predisposed to liver disease due to intracellular accumulation of Z-AAT polymers [30,31].

## 3. Clinical Phenotype of AATD-COPD

Loss of antiprotease protection due to AATD is most pronounced in the lungs, with clinical manifestations ranging from increased susceptibility to emphysema [8,32,33], COPD development [8,28,32,33,34], and, less frequently, bronchiectasis [8,32,35]. Disease onset and progression of these disorders are strongly influenced by environmental exposures, particularly cigarette smoke, as well as occupational inhalants, and recurrent respiratory infections [36,37].

The classical presentation of AATD-related COPD is characterized by the development of early (typically before 50 years of age), lower-lobe-predominant emphysema, often in individuals with minimal or no smoking history [19,38]. However, contemporary registry and imaging studies have revealed considerable phenotypic heterogeneity, including upper-lobe-predominant or mixed emphysema patterns, as well as multifaceted disease with airway involvement [8,39,40,41]. Clinically, patients may present with features overlapping those of non-deficient COPD, such as chronic bronchitis, persistent bacterial infections, frequent exacerbations, airflow obstruction with some degree of reversibility, dyspnea on exertion due to major lung inflation, and impaired health status, with disease severity often disproportionate to smoking history [42,43,44].

Physiologically, the emphysematous clinical phenotype in AATD is typically characterized by progressive decline in both forced expiratory volume in 1 s (FEV_1_) and gas transfer, reflecting small airway collapse due to loss of elastin support and alveolar integrity [8,45]. Although declines in FEV_1_ and diffusing transfer for carbon monoxide (DLCO) are generally correlated, they do not always align [8,39,40,46]. This discordance likely reflects different emphysema phenotypes, with FEV_1_ impairment more frequently being associated with basal panlobular disease and the severe Pi*ZZ genotype, while more prominent DLCO reduction is often observed in those with the less severe Pi*SZ variant and apical centrilobular emphysema (similar to non-deficient COPD) [27,39]. Interestingly, in never-smoking Pi*ZZ individuals, gas transfer impairment can be detected as early as the end of the third decade of life, preceding overt airflow limitation until the end of the fifth decade [41]. This discrepancy is also evident in cases of extremely severe disease, where the impairment in gas transfer and its progression are more pronounced, while the rate of FEV_1_ decline may be minimal [47,48]. These findings emphasize that FEV_1_ alone may be a poor surrogate for emphysema in a subset of patients with AATD-related COPD, while reduced gas transfer may serve as an early marker of parenchymal lung involvement, highlighting the importance of multimodal physiological assessment.

In their classical pathological studies, Hogg and colleagues demonstrated that loss of small airways represents an early event in COPD, preceding emphysema development and measurable airflow limitation [49]. While the trajectory of lung function decline in younger adults with AATD was previously unclear, emerging evidence has clarified its longitudinal evolution. Epidemiological studies have demonstrated the presence of early small airway pathology, analogous to that observed in non-deficient COPD, in young AATD individuals with respiratory symptoms and preserved spirometry, identifying a group at risk for subsequent disease progression [50,51]. Thus, identification of SAD may represent a potentially early marker of disease progression in AATD-related COPD, providing a potential window for intervention before extensive and irreversible structural damage occurs [52].

Bronchiectasis, a well-recognized comorbidity in non-deficient COPD, is also increasingly considered as a clinically relevant but variably expressed manifestation of the AATD-related COPD [35,53,54,55]. While early studies based on CT findings reported a high prevalence of bronchiectasis in AATD individuals with regular sputum production and frequent respiratory infections [35,56], more recent data from the European Antitrypsin Research Collaborative (EARCO) database, indicate that radiologic evidence of bronchiectasis occurs in a minority of Pi*ZZ patients, either alone (9%) or in combination with emphysema (27%) [57]. Although prevalence varies across populations, genotype studies indicate that AATD variants such as Pi*ZZ and Pi*SZ are observed in bronchiectasis in similar frequencies compared to COPD/emphysema-AATD, suggesting that AATD should be considered a relevant genetic contributor to the whole spectrum of airway diseases [55,58]. In line with these findings, current guidelines recommend screening for AATD in relevant clinical contexts, particularly when bronchiectasis is associated with emphysema or diffuse cystic bronchiectasis without clear etiology [59,60].

## 4. Diagnosis and Monitoring

**Figure 2:**
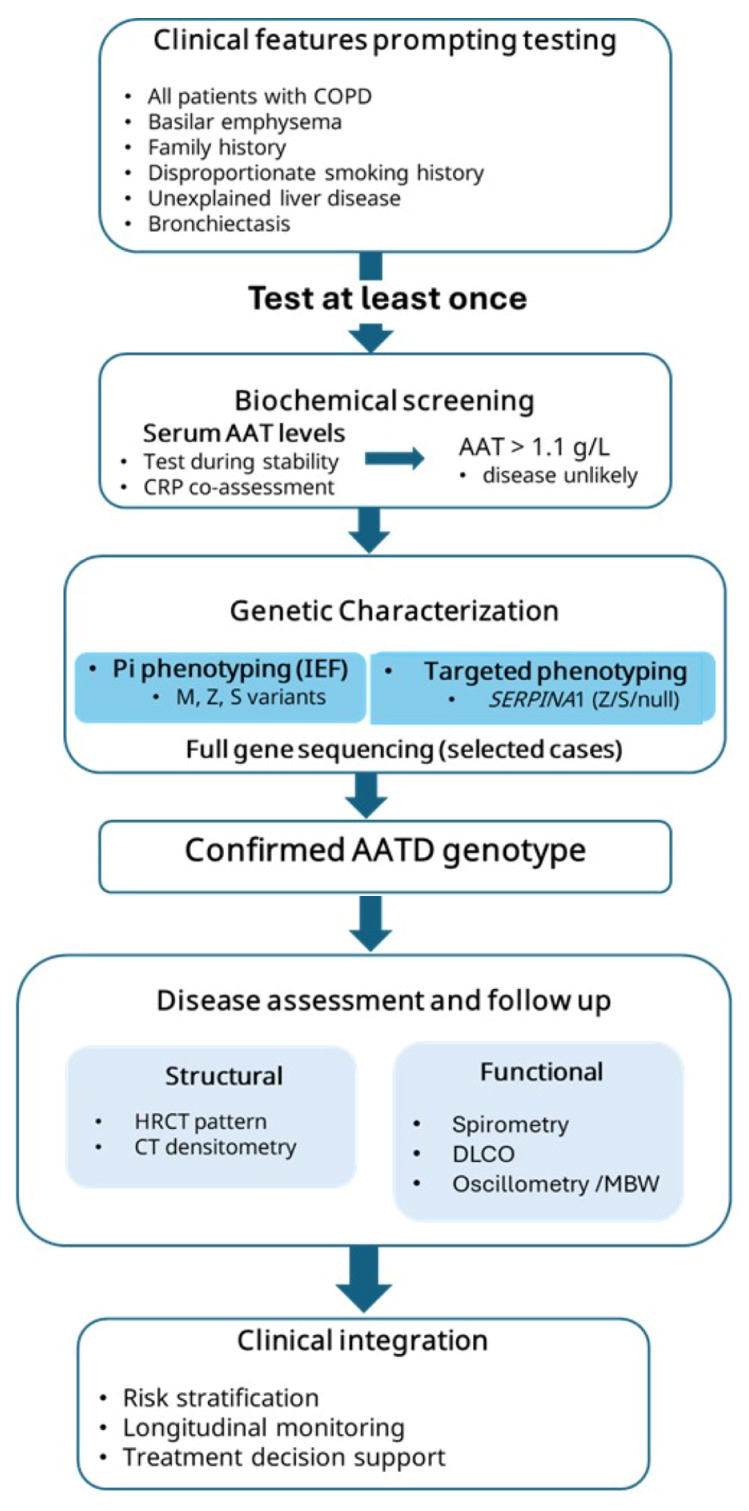
Diagnostic and monitoring algorithm for AATD-related COPD: stepwise approach from indications for initial investigation, including biochemical and genetic confirmation, with integrated structural and functional assessment to guide risk stratification and management. Created in BioRender. Fouka, E. (1 February 2026) https://BioRender.com/zxzx9bb. AATD: Alpha-1 Antirtypsin deficiency; COPD: chronic obstructive airway disease; IEF: isoelectric focusing; PCR: Polymerase Chain Reaction; HRCT: high-resolution computed tomography; DLCO: Diffusing Capacity of the Lungs for Carbon Monoxide; MBW: Multiple Breath Washout.

Alpha-1 antitrypsin deficiency represents a rare COPD endotype, accounting for approximately 1% of all COPD cases and up to 2% of those with emphysema [61,62]. Most major respiratory organizations recommend that all patients with COPD, particularly those with early-onset (<50 years) emphysema or emphysema disproportionate to smoking exposure, bronchiectasis or liver disease of unknown cause, as well as first-degree relatives of patients with AATD, should be tested at least once in their lifetimes, regardless of their smoking history [19,63,64,65]. However, despite established recommendations, the condition remains markedly underdiagnosed worldwide, with <10% of affected individuals identified [63]. Given its potential to cause preventable morbidity, early recognition is essential for risk factor modification, genetic counseling and appropriate disease management. A structured diagnostic and monitoring algorithm for AATD-related COPD, providing a stepwise approach from clinical suspicion to laboratory confirmation and family screening, is summarized in Figure 2.

Initial screening begins with the quantitative measurement of serum AAT concentration, with severe deficiency defined as AAT levels ≤0.57 g/L, and milder as levels between <1.1 g/L and >0.57g/L [19]. Alpha-1 antitrypsin is an acute-phase protein; therefore serum levels may be elevated during systemic inflammation and should be interpreted in conjunction with C-reactive protein (CRP), with testing ideally performed during clinical stability to avoid misclassification [66].

Levels below 1.1 g·L^−1^ (≈25 μM) prompt further confirmatory testing. However, serum AAT thresholds should be interpreted within the context of guideline recommendations rather than as absolute cut-offs, as diagnostic pathways may vary across practice settings, including differences in initial testing strategies and the use of reflex genotyping or sequencing. Isoelectric focusing (Pi phenotyping) allows for the identification of common variants (M, S, Z) and the differentiation between normal (PiMM), deficient (Pi*ZZ, Pi*SZ), and carrier (Pi*MZ) genotypes [67]. Genotypic testing via dried blood samples, primarily via PCR-based assays for *SERPINA1* Z and S alleles, can detect heterozygous or rare/null variants missed by phenotyping [68]. In complex cases or when clinical suspicion remains high despite inconclusive results, full gene sequencing should be considered to identify rare or uncommon variants, such as E, F, G, I and P [69,70].

Beyond biochemical and genetic confirmation, imaging plays a central role in assessing pulmonary involvement in AATD-related COPD, with conventional chest radiographs or quantitative CT scans demonstrating the characteristic basilar-predominant panacinar emphysema pattern in typical cases [71]. However, due to the various phenotypic manifestations of the disorder, absence of these findings should not preclude its existence [72]. Densitometric high-resolution CT (HRCT) is more sensitive to changes in lung density compared to qualitative CT, particularly in patients with normal spirometry, and has therefore been adopted as a primary endpoint in randomized controlled trials of augmentation therapy, reflecting its sensitivity to detect treatment effects over time [19,42]. Importantly, CT densitometry is considered a robust surrogate marker of emphysema progression, reflecting structural lung destruction more directly than spirometric indices. Changes in lung density have been associated with clinically meaningful outcomes, including mortality, supporting their relevance beyond purely radiological assessment [73,74,75]. In contrast, spirometric measures such as FEV_1_ may fail to adequately capture disease progression in AATD-related COPD, particularly in patients with predominant parenchymal destruction or discordant physiological patterns, especially in cases with early disease or in advanced stages where gas transfer decline may precede or exceed airflow limitation. These observations support the use of CT densitometry as a sensitive tool for disease monitoring, although its application should be balanced against limitations including standardization, radiation exposure, and availability, and should be interpreted in conjunction with clinical and functional parameters.

Pulmonary function testing remains central for clinical assessment and follow-up in AATD-related COPD. Similar to non-deficient disease, spirometry is used to identify patients with airflow limitation and to classify disease severity, while DLCO measurement provides complimentary information on the extent of alveolar destruction and gas exchange impairment [19,64,76,77]. However, airflow obstruction and gas transfer impairment do not always progress concurrently, therefore reliance solely on spirometry may underestimate the extent of emphysema in a subset of patients [78]. Data from the EARCO registry indicate that this discordance between spirometric and gas exchange indices is associated with distinct clinical profiles, including differences in exacerbation frequency and comorbidities [8].

Longitudinal registry studies further highlight the heterogeneity in lung function trajectories in AATD-related COPD. Data from the UK registry [42] imply that, in non-augmented AATD patients, FEV_1_ decline tends to be more rapid (>1% predicted/year) in earlier COPD stages (GOLD 1–2), although this may, in part, reflect a survivor bias, with mortality in continued rapid decliners being more likely. Notably, gas transfer tends to decline more rapidly in advanced disease, indicating ongoing emphysema progression despite relatively stable spirometry [47]. Another study from the EARCO registry revealed that never-smoking AATD patients diagnosed after the age of 65 exhibited increased spirometry and DLCO values, along with higher AAT levels, suggesting that the disorder may remain subtle or underrecognized until later in life [79].

As conventional spirometry is relatively insensitive to early-stage small airway disease, newer, functional techniques, such as oscillometry and multiple breath washout test have emerged as promising tools to detect ventilation inhomogeneities and early changes in small airway mechanics in AATD-related COPD [52,80,81]. Although not yet routinely implemented in clinical practice, these methods may help to refine risk stratification in selected patients.

## 5. Management of AATD-Related COPD

### 5.1. Standard Pharmacological and Non-Pharmacological Interventions

Management of AATD-related COPD broadly aligns with standard COPD guidelines, with disease-specific modifications. More importantly than usual, elimination of risk factors, such as smoking and exposure to fumes or mineral dusts, should be advised to reduce emphysema risk and to normalize disease progression toward rates observed in never-smokers [19,28,29,65]. In AATD-related COPD, disease progression is significantly affected by exacerbation frequency and severity, which increase disease burden by augmenting inflammation and NE activity, accelerating tissue damage similar to non-deficient COPD [82]. Timely treatment of infection with antibiotics when necessary, and recommended vaccinations (influenza, pneumococci, COVID-19, respiratory syncytial virus, pertussis and shingles) for exacerbation prevention should be key in disease management [19,65,83,84]. Optimizing inhaled therapies (long-acting muscarining antagonists [LAMA], long-acting beta-agonists [LABA] and inhaled corticosteroids [ICS]) for symptomatic patients by evaluating symptoms, exacerbation history, and blood eosinophil levels, as in usual COPD, as well as using macrolides or roflumilast in frequent exacerbators, is also advised [19,65,85]. Pulmonary rehabilitation has been shown to improve exercise capacity and QoL in symptomatic patients, although to a lesser extent than in non-AATD COPD [86]. Despite the absence of dedicated trials, long-term oxygen therapy for respiratory failure, lung volume reduction (surgical or endobronchial valves) in selected hyperinflated phenotypes, including as a bridge to transplant, and lung transplantation for end-stage disease should be discussed, following standard indications [19,65,87].

### 5.2. Augmentation Therapy for AATD

Augmentation therapy with intravenous purified human alpha-1 proteinase inhibitor (A1-PI) remains the only disease-modifying therapy for AATD-related emphysema. This therapeutic strategy was initiated in the late 1980s, following clinical trials demonstrating that weekly infusions of 60 mg·kg^−1^ restore circulating AAT levels above the proposed “protective threshold” and may attenuate lung function decline in deficient patients [74,88,89]. The rationale for treatment is based on restoring antiprotease activity within the lung, thereby limiting neutrophil elastase-mediated tissue destruction and inflammation [90].

Randomized control trials have consistently demonstrated that augmentation therapy in patients with AAT levels < 11 μM slows emphysema progression as assessed by qualitative and densitometry CT scans, supporting a disease-modifying effect on structural lung damage [73,91]. In contrast, its impact on survival remains less clearly established. Observational registry studies suggest an association with improved survival and attenuation of lung function decline [74,89,92] consistent with the prognostic significance of lung density preservation in AATD-related COPD, as well as in “classic” COPD [93]; however, these findings are subject to potential bias and confounding. Moreover, a 7-year longitudinal study failed to demonstrate a significant reduction in mortality rates, despite modest improvements in QoL [94]. Interpretation of survival outcomes is further complicated by the methodological challenges inherent to AATD, including disease rarity, heterogeneous and often slow progression, and the logistical and ethical constraints of conducting long-term randomized trials with mortality endpoints.

Based on available evidence, current international guidelines recommend augmentation therapy for non-smoking adults (<70 years of age) with confirmed severe AATD (plasma levels below protective threshold) due to Pi*ZZ genotype, and established airflow obstruction (FEV_1_ between 35 and 70% predicted) [19,64]. However, recent data question the use of a fixed biochemical threshold alone to guide treatment decisions, highlighting the importance of genotype (including individuals with the Z/null and Pi*SZ or Pi*MZ variants, which may still develop clinically significantly disease under certain conditions), clinical phenotype, environmental modifiers and longitudinal disease behavior as more reliable indicators for augmentation therapy [94]. Moreover, the initial decision for treatment based mainly on baseline spirometry alone may not adequately capture individual disease trajectories, even in patients with established COPD [42,95]. In this context, CT densitometry, which has been validated as a more sensitive and specific marker of emphysema progression and mortality risk, may serve as a complementary tool to support more individualized treatment decisions [96,97].

Despite its clinical benefits and despite its demonstrated effects on emphysema progression, augmentation therapy has important limitations. Lifelong intravenous administration imposes a substantial treatment burden, while high costs and variability in healthcare access significantly restrict its global availability and real-world implementation [98,99]. In addition, standard dosing may not fully normalize elastin degradation in all patients, requiring consideration of optimal dosing or alternative delivery routes to overcome these limitations [100,101]. Additionally, augmentation therapy is not recommended for individuals with mild or moderate deficiency due to insufficient evidence of benefit [89]. Finaly, variability in plasma-derived product characteristics further underscores the need for continued optimization and development of alternative therapeutic approaches [102].

### 5.3. Emerging and Future Therapies

Given current limitations of plasma-derived augmentation therapy, there is growing interest in novel therapeutic approaches to improve lung delivery, correct the underlying genetic defects, and prevent or reverse organ damage in affected patients.

Inhaled formulations of aerosolized AAT have been developed to enhance lung bioavailability and potentially improve convenience. Although early studies showed increased concentrations in the lung epithelial lining fluid with inhaled AAT compared with intravenous administration [103,104], a recent RCT in patients with severe PI*ZZ AATD-related COPD and frequent exacerbations failed to show a reduction in exacerbation frequency and reported more frequent treatment-related adverse events, such as dyspnea, cough, respiratory tract infection, and nausea [105]. The lack of consistent clinical efficacy despite improved drug delivery represents an important limitation to the widespread adoption of aerosolized AAT therapy. Nevertheless, an ongoing prospective phase III trial is expected to further evaluate its efficacy and safety in patients with AATD-COPD with moderate airflow limitation (FEV_1_ ≤ 50–80% predicted and FEV_1_/SVC ≤ 70% predicted) and may help clarify its potential therapeutic role [106].

Treatments targeting the mutant Z-AAT protein aim to promote correct folding and secretion, enhance degradation, and prevent intracellular polymerization. Early attempts with chemical chaperones (e.g., 4-phenylbutyric acid), small-molecule chaperones and autophagy-inducing drugs like carbamazepine have shown promise in animal models [12,107,108,109]. However, the main limitation remains the absence of robust human data demonstrating clinical benefit and clinical translation is so far limited by safety concerns.

Gene-based therapies represent a particularly attractive strategy, given that AATD is a common single-gene disorder [110]. Early phase clinical trials using recombinant adeno-associated viral (rAAV) vectors for gene transfer have demonstrated safety; however, they only achieved AAT expression levels below the therapeutic threshold, indicating the need for further optimizing vector dosing and expression efficiency [111,112]. RNA-silencing approaches targeting the mutant *SERPINA1* gene or its messenger RNA have shown significant reductions in hepatic Z-AAT accumulation, with subsequent improvements in inflammation and fibrosis [113,114]. However, this approach does not restore circulating AAT levels and therefore does not address pulmonary disease. In contrast, RNA-editing agents, such as WVE-006 and KRRO-110, aim to correct mutant *SERPINA1* gene to produce functional AAT protein, with the potential to treat both liver and lung manifestations of AATD [115,116]. DNA-level gene-editing strategies, including CRISPR-Cas-based technology, offer the prospect of permanent or long-term correction of the defective *SERPINA1* gene itself, although concerns about off-target effects remain [117]. Key challenges include achieving sustained therapeutic expression and ensuring safety, while clinically meaningful endpoints would include restoration of circulating AAT levels and prevention of lung disease progression.

Finally, novel protease inhibitors targeting protease–antiprotease imbalance and airway inflammation are under investigation and may complement disease-specific therapies in AATD-related COPD [118]. Alvelestat, an oral NE-inhibitor, has shown promising results in Phase 2 clinical trials (ATALANTa and ASTRAEUS), effectively suppressing NE activity and improving disease activity biomarkers, with a favorable safety profile [119]. However, its impact on clinically relevant outcomes such as exacerbations and disease progression remains to be established.

## 6. Expert Opinion

Alpha-1 antitrypsin deficiency-related COPD should no longer be viewed as a uniform entity defined solely as severe deficiency with the classical lower-lobe panlobular emphysema. Accumulating evidence indicates that AATD-related lung disease represents a spectrum of phenotypes shaped by genotype, environmental exposures, and functional impairment, with substantial implications for diagnosis, monitoring and treatment decision-making [19,21]. The clinical relevance of heterozygous genotypes, especially in the presence of environmental exposures such as smoking, further complicates traditional genotype-based risk assessment [10,29]. These observations argue against rigid reliance on serum AAT thresholds, supporting a more integrated evaluation that combines physiological and radiological impairment with longitudinal disease behavior [120].

A key clinical challenge lies in recognizing disease beyond advanced emphysema. Current diagnostic approaches benefit from advances in imaging and functional assessments, with CT densitometry and sensitive measures of small airway function offering superior detection and monitoring of early lung involvement insufficiently captured by conventional lung function tests [96]. Incorporation of these tools into clinical practice has the potential to identify patients at earlier, potentially more modifiable stages of disease and to improve risk stratification.

Augmentation therapy remains the cornerstone disease-modifying treatment for severe AATD-related COPD, with robust evidence supporting its role in slowing emphysema progression [19,73]. However, uncertainty persists regarding optimal patient selection, timing of initiation and clinical meaningful outcomes. The use of CT densitometry as a surrogate marker of emphysema progression [91] emerges as an alternative for treatment decisions toward more individualized treatment strategies. The clinical heterogeneity of AATD-related COPD and its implications for assessment and management are summarized in Table 1.

Emerging molecular therapies, including RNA-based interventions and gene-editing technologies, hold promise for more effective and targeted management that addresses both pulmonary and hepatic manifestations of AATD [12]. However, at the moment these strategies remain in early stages of clinical development, and their long-term efficacy and safety requires longitudinal evaluation.

Ultimately, the shift towards precision medicine in AATD-related COPD demands integration of genotype–phenotype correlations, environmental exposures, novel biomarkers, and longitudinal disease trajectories to tailor interventions. Such an approach is essential to optimize patient outcomes while translating therapeutic innovation into clinical practice.

## 7. Conclusions

In summary, AATD-associated COPD displays a broad and heterogeneous spectrum of pulmonary phenotypes, extending beyond the classic lower-lobe panlobular emphysema. Compared with smoking-related COPD, AATD-related COPD is distinguished by earlier onset, disproportional physiological impairment relative to smoking exposure, and potential extrapulmonary manifestations. Recognition of these distinguishing features is essential to prompt targeted testing for AATD for patients with COPD, especially in younger individuals and those with atypical presentations. Early diagnosis is essential as it enables appropriate risk factor modification, regular monitoring and timely consideration of disease-specific treatment strategies.

## Figures and Tables

**Table 1:** Clinical phenotypes of AATD-related COPD and considerations for individualized management.

Clinical Phenotype	Key Clinical Characteristics	Clinical Assessment	Management Considerations
Young Pi*ZZ smoker	Early-onset emphysema; often lower-lobe predominant; accelerated functional decline	Quantitative chest CT (including densitometry); spirometry; DLCO measurement; assessment of small airway dysfunction	Early consideration of augmentation therapy; strict smoking cessation and exposure avoidance; close longitudinal monitoring [19,74,75]
Older Pi*ZZ never-smoker	Mild or discordant airflow limitation; preserved FEV_1_ despite structural lung disease	Chest CT to identify emphysema–function mismatch; longitudinal DLCO and spirometric assesement	Individualized surveillance strategy; delayed or selective initiation of augmentation therapy based on disease progression [42,48,87]
Pi*MZ with COPD	Airway-predominant phenotype; variable emphysema burden; interaction with smoking and other exposures	Lung function assessment (including small airway assessment); CT-based phenotyping; careful assessment of environmental modifiers	Integrated risk assessment beyond genotype alone; tailored COPD management with selective follow-up intensity [10,29,37]
AATD with bronchiectasis	Recurrent infections; chronic sputum production; coexistence with emphysema in some patients	High-resolution CT; exacerbation frequency; microbiological surveillance	Multidisciplinary management following bronchiectasis guidelines; individualized consideration of AATD-specific implications [35,57,59,60]

## Data Availability

No new data were created or analyzed in this study. Data sharing is not applicable to this article.
